# On Characteristics of Ferritic Steel Determined during the Uniaxial Tensile Test

**DOI:** 10.3390/ma14113117

**Published:** 2021-06-06

**Authors:** Ihor Dzioba, Sebastian Lipiec, Robert Pala, Piotr Furmanczyk

**Affiliations:** 1 Department of Machine Design, Kielce University of Technology, Al. 1000-lecia PP 7, 25-314 Kielce, Poland; slipiec@tu.kielce.pl (S.L.); rpala@tu.kielce.pl (R.P.); 2 Department of Metal Science and Manufacturing Processes, Kielce University of Technology, Al. 1000-lecia PP 7, 25-314 Kielce, Poland; pfurmanczyk@tu.kielce.pl

**Keywords:** S355 steel, uniaxial tensile test, strength properties, true stress-strain relationships

## Abstract

Tensile uniaxial test is typically used to determine the strength and plasticity of a material. Nominal (engineering) stress-strain relationship is suitable for determining properties when elastic strain dominates (e.g., yield strength, Young’s modulus). For loading conditions where plastic deformation is significant (in front of a crack tip or in a neck), the use of true stress and strain values and the relationship between them are required. Under these conditions, the dependence between the true values of stresses and strains should be treated as a characteristic—a constitutive relationship of the material. This article presents several methodologies to develop a constitutive relationship for S355 steel from tensile test data. The constitutive relationship developed was incorporated into a finite element analysis of the tension test and verified with the measured tensile test data. The method of the constitutive relationship defining takes into account the impact of high plastic strain, the triaxiality stress factor, Lode coefficient, and material weakness due to the formation of microvoids, which leads to obtained correctly results by FEM (finite elements method) calculation. The different variants of constitutive relationships were applied to the FEM loading simulation of the three-point bending SENB (single edge notched bend) specimen to evaluate their applicability to the calculation of mechanical fields in the presence of a crack.

## 1. Introduction

The most fundamental test performed to define strength characteristics and plasticity of the material is a uniaxial tensile test. On the basis of this test, the basic material characteristics used in engineering methods of structural strength analysis are determined: yield strength *σ*_YS_, longitudinal elasticity modulus *E* (Young’s), ultimate tensile strength *σ*_UTS_, and plasticity characteristics-relative elongation *A* and relative necking *Z* [1,2]. The most important and most often used are Young’s modulus *E* and yield strength *σ*_YS_. The currently applied methods of strength analysis are developed based on the assumption that in the material there is a linear-elastic relationship between stress and stress (*σ* = *Eε*), and the yield strength is a quantity that limits the scope of applicability of this linear relationship (*σ* ≤ *σ*_YS_). It should be noticed that ultimate tensile strength *σ*_UTS_, plasticity characteristics: relative elongation *A* and necking *Z* play rather an auxiliary role; they enable one to qualitatively assess which material is stronger or more plastic.

However, in the case of strength assessment of components containing crack-like defects, high-stress concentration is observed near the crack tip, the intensity of which reaches several times the values of the yield strength. Moreover, the size of the plastic zone significantly exceeds the one permissible as specified in the requirements of linear fracture mechanics. In this situation, while performing strength analysis, it is essential to apply the model of the non-linear material and defined the relationship between true stress and strain values.

Defining the relationship between true stress-strain, also called the constitutive equation, requires establishing the critical stress and strain of the material. Different methods used to define such relationships were analyzed in the articles [3,4,5,6]. However, in summary, the authors of the paper [4] came to the conclusion that, up to date, no uniform and unequivocal method for determining the true stress-strain relation of the material was suggested.

To create a true stress-strain relationship of the material, the data from the uniaxial tensile test are needed. Until the specimen reaches the maximum strength (until necking starts), there is a uniform elongation of the testing coupon of the specimen, and true stress and strain are calculated building upon nominal values based on the Equation (1):

(1) $$\varepsilon_{t}=\ln\left(1+\varepsilon_{n}\right);\;\sigma_{t}=\sigma_{n}\left(1+\varepsilon_{n}\right)$$

During neck formation, the material is deformed unevenly, and different levels of stress and strain occur in various cross-sections of the specimen; the maximum values are in the minimum cross-section of the neck. To determine stress and strain in the minimum cross-section of the neck and, based on their basis, define a relationship of true stress-strain, different methods were developed and proposed.

The approach based on the correlation of stress level in the neck bottom with the use of Bridgman’s equation [7] does not enable the assessment of the plastic strain and does not involve material hardening. The extrapolation of the stress-strain relationship obtained as a result of fitting the true stress and strain values by power function over the section of uniform elongation the specimen is frequently applied. The above-mentioned approach is also not suitable since the true stress-strain relationship during necking is described rather by a linear function, not by a power function. More information on this topic is provided in the further part of this paper. Methods for establishing the true stress-strain relationships based on the iterative adjustment are also in use. In this approach is believed that the relationship is true when the numerically calculated load curve of the specimen is compatible with the diagram obtained during the uniaxial tensile test [8,9,10,11,12].

In the method of defining the constitutive relationship of a material developed by Bai and Wierzbicki [13,14,15], it was proposed to take into account the influence of characteristic values of the stress field-the stress triaxiality coefficient, Lode coefficient, and the plastic strain of a material. In the articles of Neimitz et al. [16,17,18], some modifications of the Bai and Wierzbicki method were introduced. They allowed for taking into account the emerging inhomogeneity of the material in the areas of high levels of plastic deformation.

The knowledge of constitutive relation is necessary at determining the mechanical fields in elements containing the crack-type defects or sharp notches, where are high level of concentration stress and strain. The obtained values of strain and stress distributions will depend on the correct determination of the material constitutive relationship, which has an impact on the assessment of strength and safety using of the elements [19,20,21,22].

The problem of the influence of material inhomogeneity on the process of its destruction is also analyzed in the model known as GNT (Gurson-Needleman-Tvergaard) [23,24,25,26,27,28]. In the GTN model, the process of void nucleation—growth—coalescence is directly taken into account using certain postulated functions, but the functions must be properly calibrated by experimental and numerical testing.

This article presents experimental and numerical research, the main goal of which was to determine the constitutive relationship between the true strain and stress of the material. The paper addresses test results obtained by the authors on S355 steel of ferrite-pearlite microstructure, although similar results were obtained for other types of ferritic microstructure [29,30]. The results concern the application of different test methods to provide the true stress-strain relationship, including evaluation of the specimen cross-section reduction using the voltage potential change method and video recording; microstructural and fractographic tests carried out with a scanning microscope. Numerical modeling and calculation of stress and strain fields at loading specimen containing crack (SENB) were performed for verification of the constitutive relations correctness.

## 2. Materials and Research Methods

**Figure 1:**
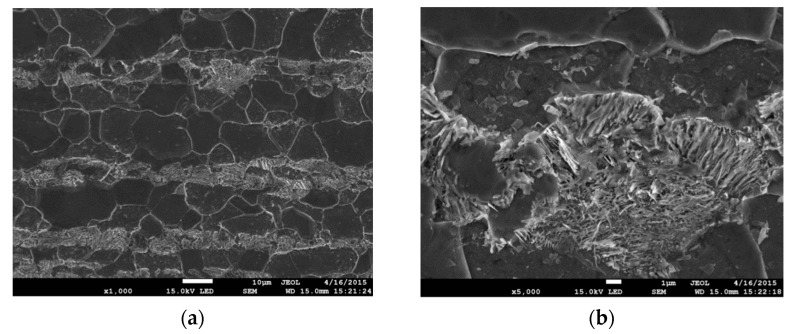
Steel S355 of ferrite-perlite (*FP*) microstructure: (**a**) ×1000; (**b**) ×5000.

The material used in tests is S355 steel (former symbol—18G2A steel). It is a structural steel similar to ASTM A765. The chemical composition of S355 steel is presented in Table 1 [31]. It is a low carbon structural steel with a medium level of strength characteristics and suitable weldability. The steels of this grade are widely applied to construct different types of building structures, tanks, and pipelines [32,33]. A laboratory heat-treatment was conducted on specimen sections to reduce the influence of the thermomechanical treatment used during the production of elements in steel plants. The specimens were normalized by annealing it for 20 min at the temperature of 950 °C, and next cooled in the air. Due to such processing were obtained of ferrite-pearlite microstructure (FP), with grain size of 7–20 µm (Figure 1), in the specimens.

The uniaxial tensile test in laboratory conditions (at 20–22 °C) was conducted on standard five-fold cylindrical specimens with an initial diameter *d*_0_ of 5.0 or 10.0 mm. Tests were performed with the use of a Zwick-100 testing machine (ZwickRoell, Ulm, Germany) with an electrodynamic drive, equipped in the automated system of loading control and data recording in real-time. The signals of the load force and elongation of the measuring distance were recorded in all tested specimens. In addition, in order to determine the minimum diameter of the specimen, video recording of the neck zone was performed during its elongation, as well as the potential of voltage change [34,35]. The details of this research would be presented in the next chapters of this article. In addition, to define the changes in the microstructure by different strain levels, some tested specimens were subjected to metallographic and fractographic testing on a scanning electron microscope (SEM, JEOL, Zaventem, Belgium) JSM-7100F. Tensile specimens and specimens containing cracks loaded by three-point scheme bending (SENB were also modeled and analyzed using the program for numerical testing ABAQUS (ver. 6.12-2, 3DASSAULT SYSTEMES, Vélizy-Villacoublay, FR-78, France).

## 3. Experimental Research

**Figure 2:**
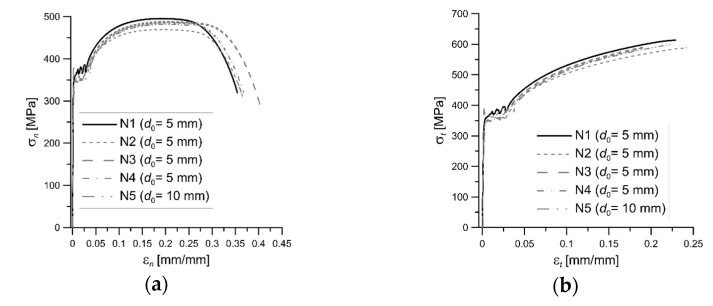
The stress-strain plots for specimens of S355 steel: (**a**) nominal values; (**b**) true values.

Nominal stress-strain curves with Luder’s yielding strain plateau were obtained for tested S355 steel (Figure 2). Based on nominal values, true stress and strain values were calculated in the range of uniform elongation (Equation (1)), and they were fitting by the power function, Equation (2):

(2) $$\sigma_{t}=\alpha \cdot {\left(\varepsilon_{t}\right)}^{n}$$

The appropriate values of strength characteristics and plasticity obtained based on nominal and true data are present in Table 2.

### 3.1. Assessment of Material Strain

**Figure 3:**
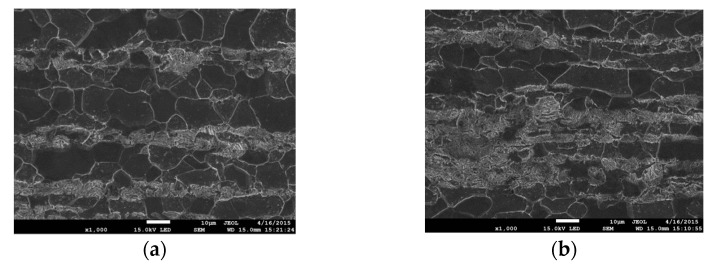

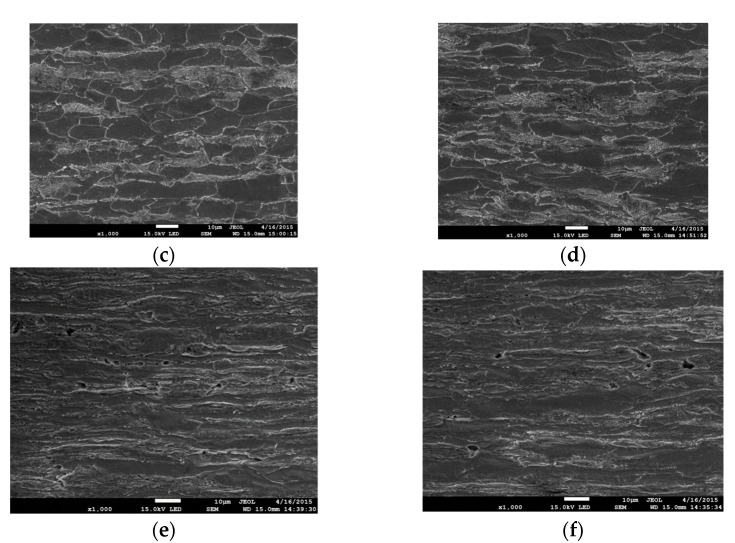
Deformed microstructure of S355 steel at different distance from the fracture plane: (**a**) 17 mm (undeformed material); (**b**) 7 mm; (**c**) 4 mm; (**d**) 2 mm; and (**e**,**f**) 0.1–0.2 mm.

Metallographic examinations were conducted on specimen sections made in an axis plane, which was polished and etched with a solution of 3%HNO_3_. The aim of this research was to determine the changes in the strain-induced material microstructure. The microstructure was observed along the specimen axis at a different distance from the fracture plane. The exemplary images taken at different distances from the fracture surface are shown in Figure 3. The images provided clearly saw the differences in the material microstructure. With the approaching proximity to the specimen fracture plane, the grains become more stretched out in the direction of specimen tensile force.

**Figure 4:**
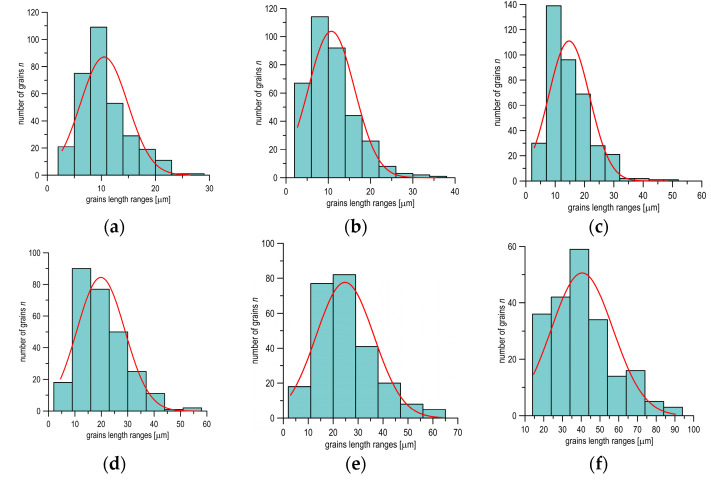
Histograms and graphs for normal distributions of grain length at different distances from the fracture plane of the specimen: (**a**) 17 mm; (**b**) 10 mm; (**c**) 5 mm; (**d**) 3 mm; (**e**) 2 mm; and (**f**) 0.1 mm.

It is easy to notice that the grains elongation near the fracture plane is several times bigger as compared to the material undeformed in the gripping section of the specimen. Grains measurement was performed on the microstructure images obtained at the appropriate distances from the fracture in order to determine the quantitative values of deformed material. The measurements were conducted in accordance with the norm requirements [36]. The statistical analysis of data was performed next. The exemplary histograms of the grain length of the microstructure of S355 steel in different distances from the fracture plane and the statistical normal distribution of grain size are shown in Figure 4.

**Figure 5:**
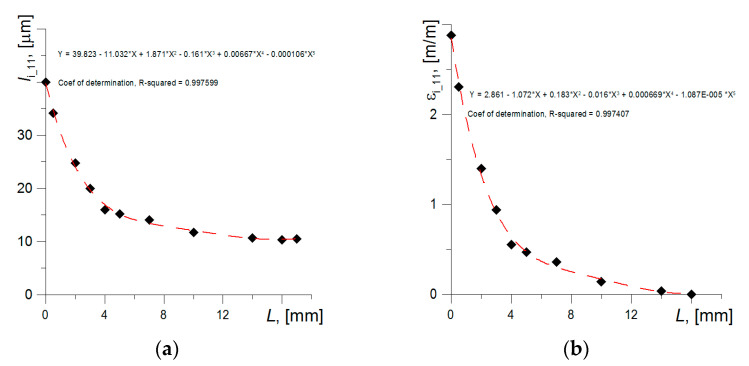
Graphs of changes in ferrite grain length (**a**) and strain level (**b**) in a uniaxially tensile specimen made of S355 steel.

The average grain size in the appropriate distance from the fracture plane was determined based on the statistical normal distribution (Figure 5a). The knowledge of the average grains size enabled to define the grain deformation at appropriate distances from specimen fracture plane. Strain in the appropriate point was calculated as: *ε*_i_11_ = (*l*_i_11_ − *l*_0_11_)/*l*_0_11_, where *l*_0_11_ and *l*_i_11_—are average grains size in the undeformed and deformed state. The strain distribution depending on the distance from the fracture plane, which was obtained based on grain measurements, is shown in Figure 5b. Strain achieves the maximum value directly near the fracture plane, so that is why the strain level in this region was assumed to be critical. Based on the conducted measurements—*ε*_c1_11_ = 2.80–3.10 = 280–310%. In a similar way, strains in the perpendicular direction (radial) *ε*_i_22_ and critical value were determined as *ε*_c_22_.

The strain level also can be determined with the assumption that the strain is the ratio of the increase in ΔΔ*L*_i_ to the length of the measuring section Δ*L*_i0_, in the case when the length of the segment Δ*L*_i0_ tends to an infinitely small value *d*:

(3) $$\underset{\Delta L_{i0}\to d}{\varepsilon =\lim}\left(\frac{\Delta \Delta L_{i}}{\Delta L_{i0}}\right)$$

where: ΔΔ*L_i_* = Δ*L_i_* − Δ*L*_*i*0_; Δ*L*_*i*0_—length of the testing segment before tensile test, Δ*L_i_*—length of the testing segment after tensile test; *d*—this value can be equated with the microstructure size, namely the size of the grain.

**Figure 6:**
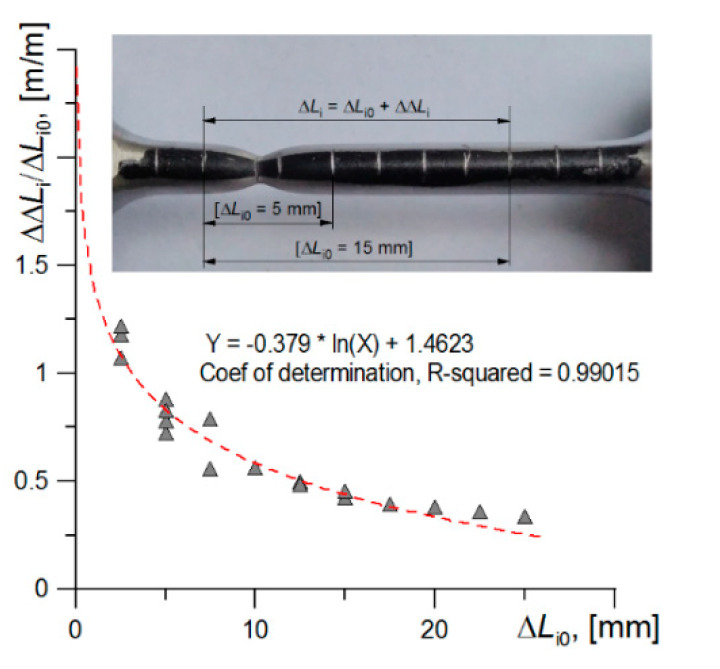
The scheme presents the measurement of strain in a uniaxial tensile test of the specimen (in the photo) and the obtained strain values together with the fitting function.

In this approach, the critical level of strain, *ε*_c2_11_, was determined by extrapolating the fit function to the size of the deformed ferrite grain. In this approach, the measuring segment of the tested specimen was divided into equal sections by plotting marks, every 2.5 mm, as is illustrated in Figure 6. When the test was performed, the elongation of each section was measured, and the ratio of elongation to the initial length of sections of various lengths ΔΔ*L_i_*/Δ*L*_*i*0_ was determined and presented the dependence of these ratios to the length of the initial sections (Figure 6). For example, the photo shows two sections with initial lengths Δ*L*_*i*0_ = 5 mm and 15 mm. The next extrapolation of the fit function to the ferrite grain size level (40–42 μm) allows us to estimate the critical deformation value: *ε*_c2_11_ ≈ 2.88.

So, the two approaches presented above are lead to obtaining similar critical strain values from the range of: *ε*_c_11_ = 2.80–3.10.

### 3.2. Assessment of the Actual Minimum Diameter of Specimen

**Figure 7:**
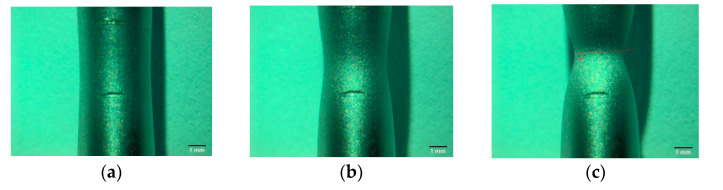
Examples of forming neck during specimen tensile (recorded by video-camera Olympus): (**a**) ~250 s, start of necking; (**b**) ~350 s; and (**c**) ~450 s, before specimen fracture.

During the uniaxial tensile test, the minimum cross-sectional area in the neck permanently changes. To calculate the actual stress in the specimen at uniaxial tensile, it is necessary to know the area of the minimum cross-section of the neck. Two methods were used for this purpose. In the first, video recording of the tensile process was used to record the size of the minimum diameter in time (Figure 7). That allowed to calculate the value of the actual stresses *σ*_a_ as the ratio of the actual value of the force to the actual value of the minimum cross-section area *S*. The stress value at the moment of specimen fracture, determined by this method, is *σ*_c1_ ≈ 1275–1300 MPa.

**Figure 8:**
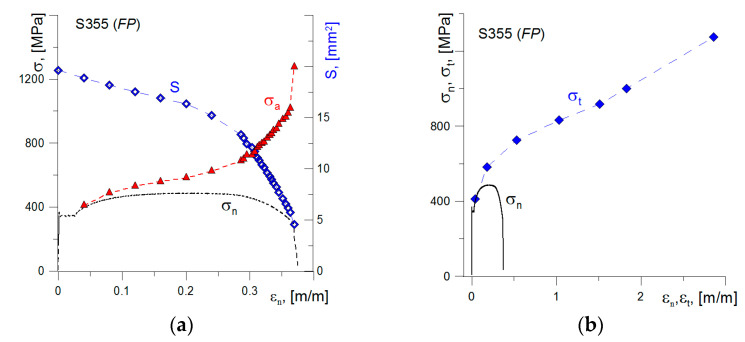
(**a**) Reduction in the specimen cross-sectional area S and increase in stresses *σ*_a_ during loading; (**b**) the stress-strain plots for nominal and true values.

Using video recording also allowed us to establish the elongation of the measured sections in time. On the basis of these measurements, the actual strain values occurring in the specimen tensile process were estimated. This allowed us to present the stress-strain diagram for true values, assuming that *σ*_a_ = *σ*_t_ (Figure 8). Based on these results, the stress-strain relationship for actual values on the neck forming section can be described by a linear function.

**Figure 9:**
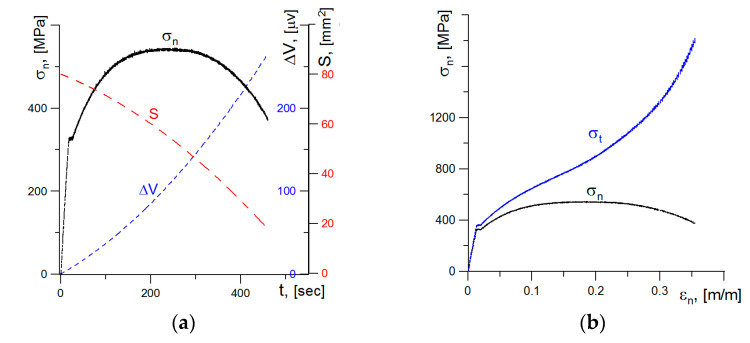
(**a**) The graphs of nominal stresses, potential increase, and minimum specimen cross-section during the tensile test; (**b**) the graphs of nominal and true stresses as a function of nominal strain.

The second method used to estimate the minimum cross-sectional area was the method of recording the change of the electric voltage potential measured on the tested sample section. This method is often used for determining length or increment of cracks growth in quasi-static or fatigue tests [34,35]. The relationships between the change in nominal stresses (*σ*_n_*-t*) and the potential value (Δ*V-t*) over time are shown in Figure 9a. When the specimen is tensile, the values Δ*V* significantly increase and is inversely proportional to the specimen minimum cross-sectional area *S*.

The stress diagram *σ*_a_ obtained as the ratio of the actual force values to the minimum cross-sectional area is shown in the Figure 9b. The true stresses increase during specimen tensile and reach a critical value at the moment of specimen breaking at the level of *σ*_c2_ ≈ 1870–2000 MPa. These values are higher as compared to those obtained via the method for determining cross-sectional area with video recording—*σ*_c1_ ≈ 1275–1300 MPa. The reason for this difference will be analyzed in the further part of this article.

### 3.3. Numerical Analysis of Stress-Strain State

The results obtained in the experimental tests followed the creation of stress-strain relationships of the tested material. The use of various research methods has led to the presentation of several different variants of the stress-strain relationship of a material. The constitutive stress-strain relationships were defined on the basis of the obtained characteristic values *ε*_c_ and *σ*_c_. The correctness of the material relationship was verified by entering it into the numerical model of the specimen that was subjected to the uniaxial tensile test. The result, in the form of the force-elongation relationship of the specimen, obtained by calculating with the use of the finite element method (FEM), was compared with the relationship recorded during the test. The suitable compatibility of graphs obtained by calculation and experimental test should indicate a correctly defined material constitutive relationship.

#### 3.3.1. Numerical Modeling

**Figure 10:**
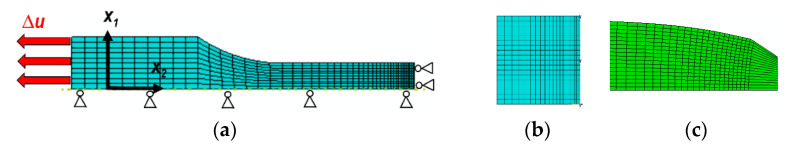
Numerical model of the tensile specimen (**a**); undeformed mesh fragment near blocked plane (**b**); deformed mesh fragment near blocked plane (**c**).

The numerical modeling of specimens was performed in the ABAQUS program. Due to the fact that in the uniaxial tensile specimen, an axisymmetric state occurs, the model of ¼ specimen, which is shown in Figure 10a, was used for the calculations. The load was applied by displacement of the plane of the specimen grip according to those recorded during the experimental tests (Figure 10a). The four-node finite elements were used in the mesh. The mesh was condensed along with the approaching to the blocked radial edge. The selection of the size of finite elements and the change in mesh condensation was preceded by initial calculations in order to obtain the convergence of the results. An enlarged fragment of the concentrated undeformed mesh near the blocked plane is shown in Figure 10b, and the same deformed fragment, in Figure 10c.

**Figure 11:**
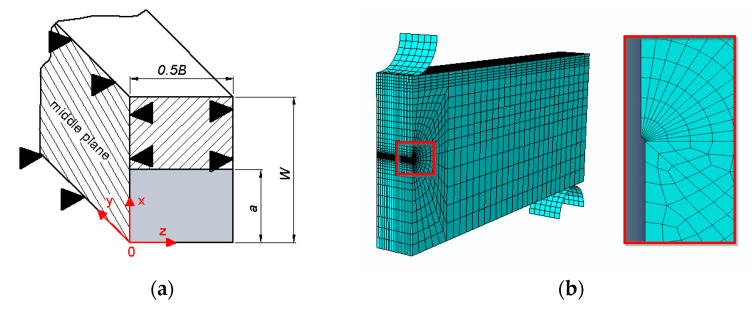
Numerical model of SENB specimen: (**a**) scheme of the boundary conditions; (**b**) scheme of the mesh.

Numerical calculations were performed to determine the stress and strain distributions in front of the crack tip. The FEM calculations have been realized on the numerical model of the SENB (single edge notched bend) specimen by using the Abaqus program. The dimensions of the SENB specimen were as follows: *W* = 24 mm, *S* = 96 mm, *B* = 12 mm, *a*_0_/*W* = 0.55. The 1/4 of the numerical specimen was modeled due to the occurrence of symmetry. The SENB specimen was divided into 21 layers in thickness direction. The eight-node three-dimensional elements were used in the calculation. The possibility of transference was blocked according to the scheme shown in Figure 11 (enabling the movement of the cracked part of the XOZ specimen, blocking the possibility of movement of the uncracked part of the XOZ specimen along the y-axis, blocking the possibility of moving the central plane of the XOY specimen along the z-axis, blockage of the lower roller). The density of the elements net increases in the direction to crack tip. The crack tip was modeled as an arc of 0.012 mm in the radius. The selection of the finite element size and the partition of the specimen into layers was preceded by preliminary analyses in order to achieve convergent analysis results with the appropriate quality of the finite element mesh.

#### 3.3.2. The Stress and Strain Distributions in the Neck of the Tensile Specimen 

**Figure 12:**
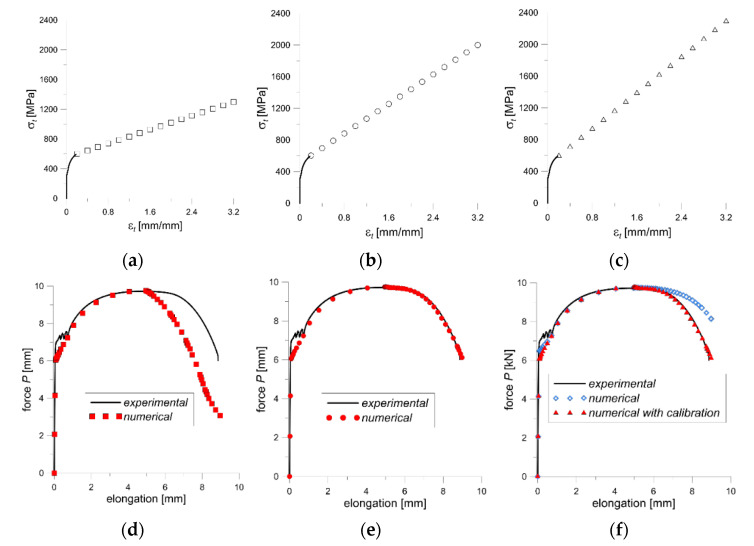
The stress-strain graphs for: (**a**) var. 1, (**b**) var. 2, (**c**) var. 3, and suitable numerical and experimental force-elongation curves for: (**d**) var. 1, (**e**) var. 2, and (**f**) var. 3.

Three variants of the material stress-strain relationships were investigated in a numerical simulation. The constitutive relationship used as the first variant (var. 1) is presented by a stress-strain diagram with critical values: *ε*_c_ = 3.2 and *σ*_c_ = 1300 MPa (Figure 12a). For the second variant (var. 2), a constitutive relationship was assumed for the critical value of stress at *σ*_c_ = 2000 MPa and strain of *ε*_c_ = 3.2 (Figure 12b). The calibration method of the constitutive dependence of material suggested by Bai and Wierzbicki [13,14,15] with the modification of Neimitz et al. [16,17] was also verified (var. 3; Figure 12c). In the Bai and Wierzbicki method, the influence of the stress triaxiality factor in the specimen is taken into account during calibrating of the material constitutive relationship. In turn, the modification proposed by Neimitz takes into account the weakening of the material at the expense of the formation of voids during the neck formation. These suggestions will be further talked over in the Discussion chapter.

The comparison of the curves obtained experimentally and the calculated by FEM for the tensile-loaded specimens are shown in Figure 12d–f. The curve FEM calculated is lower than the experimental one in an interval corresponding to the necking zone for var. 1, Figure 12d. The difference between these curves increases with the necking intensification. Thus, the obtained result indicates that the constitutive relation (var. 1) is not properly.

For var. 2 and var. 3, the experimental and computational curves are very close to each other, and they almost overlap (Figure 12e,f). This means that the constitutive relations with the proposed parameters are correct.

**Figure 13:**
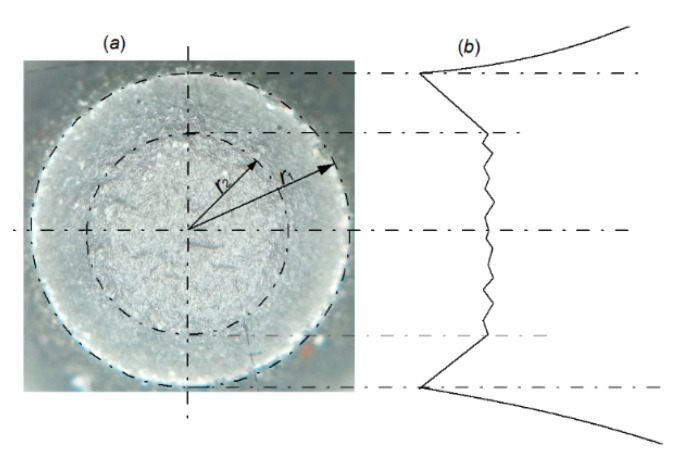
View of break surface of the tensile specimen (**a**) and a scheme of the break plane profile (**b**).

An important aspect, which should be taken into consideration, is the character of fracture surface in a specimen subjected to the uniaxial tensile test. The specimen break surface shows two areas, which differ in fracture mechanism (Figure 13). In the central part of the fracture surface is observed zone with radius *r*_2_ called the “cup bottom”, which is perpendicular to the specimen axis. That zone consists of many small areas where the fracture process developed mainly according to the void growth and shear mechanisms. While, near the side surfaces of the specimen, the crack development follows along quasi-conical surface mainly according to the shear mechanism. The outside radius (*r*_1_) measured in the neck includes these two areas.

The critical stress level calculated as the force value at fracture to the surface of the inner region leads to the results in the range of 2100–2300 MPa, which is higher than the critical stress value obtained by the method for changing the voltage potential (1870–2030 MPa). Direct application of such a relationship in numerical calculations leads to obtaining values higher than the experimental ones (blue symbols in Figure 12c). However, carrying out calibration taking into account the state of stresses and strains as well as the material weakening (var. 3) leads to the obtaining of computational results consistent with the experimental ones (red symbols in Figure 12c).

#### 3.3.3. Stress and Strain Analysis in Crack Front of the SENB Specimen 

Calculations of mechanical fields for the model of the SENB specimen also were performed in order to check the correctness of the results obtained by using different constitutive relations (var. 1, var. 2, and var. 3). For the select point of the specimen deflection (Δ*u* = 1.2 mm), the distributions of the stress and plastic strain in front of the crack tip are presented for the symmetry plane of the SENB specimen, where the highest values occurred (where: *σ*_11_—in the direction of crack growth, *σ*_22_—in the direction of perpendicular to the crack plane, *σ*_33_—in the direction of the specimen thickness). The stress component *σ*_22_ and plastic strain *ε_pl_* have a dominant role in fracture process initiation [37,38,39,40,41].

**Figure 14:**
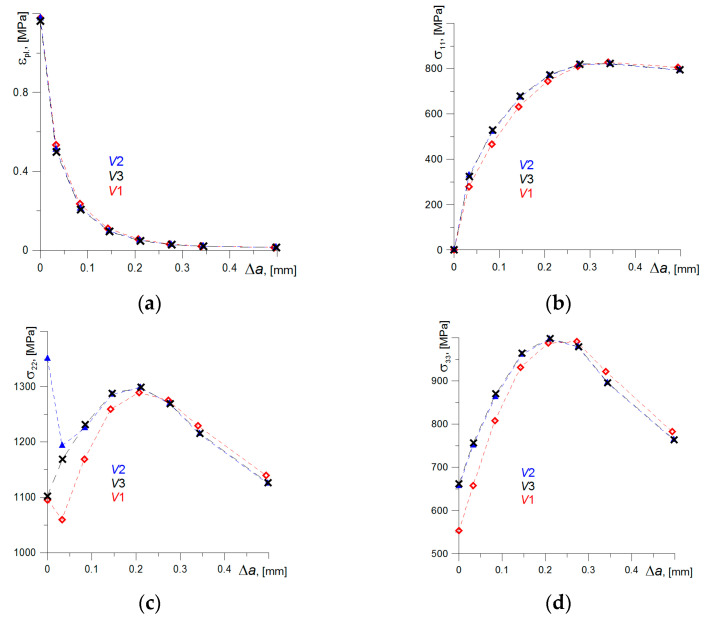
The distributions of plastic strains (**a**) and of stress components (**b**–**d**) in front of the crack calculated by means of FEM.

In plastic strain distributions *ε_pl_*, the differences are insignificant, while in stress distribution, they are visible (Figure 14). It is clearly noticeable that stress components obtained using a constitutive relationship of var. 1 demonstrate a significant deviation from the results computed of var. 2 and var. 3, especially in of *σ*_22_ and *σ*_33_ distributions (Figure 14c,d). In the vicinity of the crack tip, where the highest strain levels are observed, the differences are 100–150 MPa. While receding from the crack tip and along with the decrease in the strain level, the difference between stress distributions determined by assuming various constitutive relations is on the decrease. Thus, the using the constitutive relation of var. 1 in calculations leads to obtaining incorrect stress values, especially in high-strains zone near the crack tip.

The stress distributions *σ*_22_ and *σ*_33_ were calculated according to constitutive relationships of var. 2 and var. 3 overlap everywhere except the area near the crack tip. The differences in stress distribution curves are observed for *σ*_22_ stress (Figure 14c). They are due to different assumptions in defining constitutive relations in var. 2 and var. 3. This problem will be discussed in detail in the Discussion chapter below.

## 4. Discussion

The comparison of the results obtained according to FEM with the experimental results shows that for the correct numerical mapping of the specimen load, the proper definition of the stress-strain relationship of the material is of key importance. However, only the verification carried out using FEM calculations allows indicating the proper of constitutive relation.

The results received according to the methods for determining the constitutive relationship for var. 2 and var. 3 are similar to each other, although they are based on different approaches. In the var. 3 method, developed by Bai and Wierzbicki [13,14], the constitutive relationship is defined based on the characteristics of the stress field, Equation (4):

(4) $$\sigma_{yld}=\bar{\sigma }({\bar{\varepsilon }}_{p})\left[1-c_{\eta }\left(\eta -\eta_{0}\right)\right]\left[c_{\theta }^{s}+\left(c_{\theta }^{ax}-c_{\theta }^{s}\right)\left(\gamma -\frac{\gamma^{m+1}}{m+1}\right)\right]$$

where:

- $\bar{\sigma }({\bar{\varepsilon }}_{p})$—the function that describes the true stress-strain relationship, which is obtained experimentally during the tensile test;
- *η*—triaciality stress factor: $\eta =\frac{\sigma_{m}}{\sigma_{e}};$$\sigma_{m}=\frac{1}{3}\left(\sigma_{xx}+\sigma_{zz}+\sigma_{yy}\right)$; $\sigma_{e}$—effective stress; $\eta_{0}$—the reference factor of the triaxial stress factor in the calibrated specimen (for uniaxial tensile specimen—0.33);

Quantities $c_{\eta }$, $c_{\theta }^{s},\;c_{\theta }^{ax}$, m are usually determined experimentally. Quantities $c_{\theta }^{s},\;c_{\theta }^{ax}$ are related to the Lode angle value: *θ* ($c_{\theta }^{ax}$ = $c_{\theta }^{t}$ for *θ* ≥ 0, $c_{\theta }^{ax}$ = $c_{\theta }^{c}$ for *θ* < 0);

The function *γ* is calculated from the Equation (5):

(5) γ=6.46[sec(θ−π/6)−1]

Lode angle is calculated from the Equation (6):

(6) $$\bar{\theta }=1-\frac{6\theta }{\pi }=1-\frac{2}{\pi }arccos\xi$$

where:

(7) $$\xi =L\frac{9-L^{2}}{\sqrt{{\left(L^{2}+3\right)}^{3}}}$$

where *L* is the Lode parameter, calculated from the formula:

(8) $$L=-\frac{2\sigma_{II}-\sigma_{I}-\sigma_{III}}{\sigma_{I}-\sigma_{III}}$$

where: *σ_I_* is the biggest and *σ_III_* the smallest component of principal stress.

In articles Neimitz et al. [16,17,18], modification of the factor *c*_η_ was suggested, which considered the increasing effect of material weakness along with the increased level of in high plastic strain, Equation (9):

(9) $${c_{\eta }}^{\prime }=c_{\eta }{\left[1+H\left(\varepsilon_{pl\_0}\right)\left(\varepsilon_{pl\_i}-\varepsilon_{pl\_0}\right)\right]}^{\alpha }$$

where:

$\varepsilon_{pl\_0}$—plastic strain level, by which the onset of void coalescence takes place;

*α*—the exponent takes values in the range from 5.0 to 6.0;

*H*($\varepsilon_{pl\_0}$)—is a Heaviside function.

**Figure 15:**
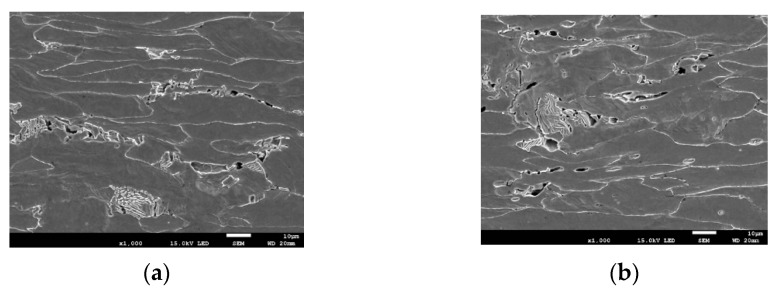
Development of voids in a uniaxially tensile specimen: (**a**) ~1.0 mm since the fracture plane; (**b**) directly at the fracture plane.

The process of nucleation, growth, and coalescence of voids in the material was confirmed by SEM tests of microsection of specimen axial plane after uniaxial tensile (Figure 15). The increase in the number and the size of voids was observed while approaching the plane of specimen fracture plane, where the plastic strain increases, which qualitatively confirms the correctness of modification introduced by Neimitz et al. [16]. A detailed description of determining the quantities used while defining the constitutive relation according to var. 3 on specimens made from S355 steel with different geometrical shapes was described in papers [17,18].

FEM results similar to var. 3 were obtained using the stress-strain relationship defined on the basis of determining the minimum cross-section of the specimen using the change in voltage potential (var. 2). On the other hand, the results obtained by applying the stress-strain relationship for var. 1, which was defined based on the data of the specimen outside diameter, differ significantly from data for var. 2, var. 3, and of the experiment. Such a result indicates that the true stress determined based on the measurement of the outside minimum diameter in the neck of the specimen is incorrect.

The stress triaxiality factor, *Lode* coefficient, and effect of material weakness in high-strain zones were taken into consideration when determining the constitutive stress-strain relationship for var. 3. However, when defining a constitutive dependency for var. 2 indirectly, only the material weakening effect has been taken into account, while changes in the stress triaxiality factor and *Lode* coefficient are not taken into account. The impact of the stress triaxiality factor and *Lode* coefficient is revealed at high-strain levels when the plastic fracture mechanism occurs through the growth and coalescence of voids; thus, the differences in stresses are recorded for true plastic strains *ε_pl_* ≥ 0.4.

The calculation of stress distributions by means of FEM indicates that the use of the constitutive stress-strain relationship according to var. 3 leads to obtaining the correct results in the entire diapason of strain values the material. In the case of using the constitutive relationship according to var. 2, the results convergent with var. 3 were received for range *ε_pl_* < 0.4, which corresponds to strain up to the beginning of the neck formation for the specimen uniaxial tensile.

**Figure 16:**
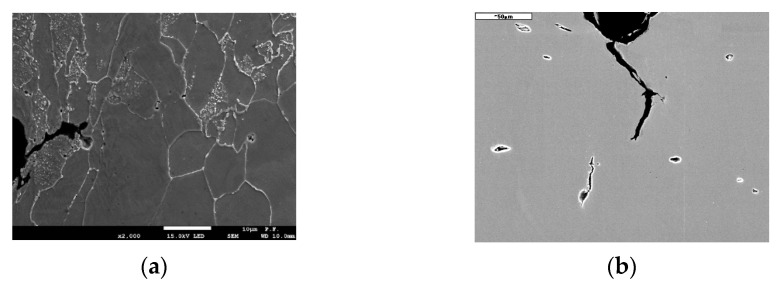
Deformed and damaged material in front of crack tip in SENB specimen: (**a**) S355 steel; (**b**) 14MoV6 steel.

The presented approach to defining the constitutive relationship of the true stress-strain of the material allows for the analysis of mechanical fields in areas with very high levels of plastic strains. In tests carried out by the authors, the material weakness effect due to the voids growth was noticed in different types of ferritic steels in tensile and bending tests (Figure 15 and Figure 16) [17,30,42]. The presented method, as well as the GNT method [23,24,25,26,27,28], allow for the accurate analysis of mechanical fields in areas with very high levels of plastic strain, where the material is no longer actually homogeneous. The problem related to carrying out numerical calculations and determining the distribution of mechanical fields before the tip of crack-type defects is a very important aspect on which the local approach to the analysis of the strength of structural elements is based [43,44,45,46,47].

## 5. Summary

This article presents the procedure of defining the true stress-strain relationship. For this purpose, it is necessary to take into account the coefficient of stress triaxiality, *Lode* coefficient, and the phenomenon of material weakening as a result of the development of microvoids. In addition, there is necessary to determine a key material characteristic—critical values of strain and stress. To determine the constitutive relationships of steel S355 during tensile loading, the following characteristics should be received.

**Material critical strain value—*ε_c_*.** This quantity can be obtained based on the methods described in the article.

- According to the metallographic method based on strain measurement of the material microstructural component—grains;
- According to the method basing on the extension measurement of the decreasing segment of the specimen with the further extrapolation of the fit function to the level corresponding to the grain size of the material. Applying video recording during the uniaxial tensile test enables us to estimate true strain values for each moment of neck formation and not only for the critical when fracture occurs.

Both of these methods can be simultaneously applied during uniaxial tensile on the same specimen. The critical strain level evaluated by using the above-mentioned methods is obtained as an estimated value, thus adopting various approaches allows us to compare the result and its supplement and increase the probability of correctness.

**Material critical stress value—*σ_c_.*** The article presented a few methods of determining this quantity and carried out verification of its correctness.

- Seemingly the simplest—the force divided by the full cross-section in fracture moment of the specimen does not lead to obtaining the correct material constitutive relationship, which has been confirmed by verification performed by FEM calculations;
- The critical stress values obtained based on the change electric voltage method allow us to obtain the material constitutive relationship, which leads to convergence of the experimental and calculated force-displacement relations.

The use of the constitutive relationship defined by method, which takes into account the impact of high plastic strain, the stress triaxiality factor, Lode coefficient, and material weakness due to the growth of microvoids, allowed proper results in numerical modeled uniaxial tension test and in the test of bending of the specimen with crack (SENB).

## Figures and Tables

**Table 1:** Chemical composition of S355 steel (weight %).

C	Si	Mn	Cr	Ni	S	P
0.18	0.2–0.5	1.5	max. 0.003	max. 0.003	max. 0.004	max. 0.004

**Table 2:** The strength and plasticity properties of S355 steel.

S355 Steel	*σ*_YS_L_, (MPa)		*σ*_YS_H_, (MPa)		*σ*_UTS_, (MPa)		*E*, (GPa)		*n*		*A*_5_, (%)
	Nom.	True	Nom.	True	Nom.	True	Nom.	True	Nom.	True	Nom.
Average	366.7	368.3	377.5	380.0	489.6	596.9	200	201	7.89	4.78	37.23
Maximum	375.7	379.5	381.9	392.0	495.6	613.3	203	204	8.93	5.08	40.45
Minimum	353.3	356.7	357.6	362.7	479.6	587.9	198	198	6.96	4.28	35.12

## Data Availability

Data available on the request to the correspondence author.
